# Development of a clinical prediction model for the onset of functional decline in people aged 65–75 years: pooled analysis of four European cohort studies

**DOI:** 10.1186/s12877-019-1192-1

**Published:** 2019-06-27

**Authors:** Nini H. Jonkman, Marco Colpo, Jochen Klenk, Chris Todd, Trynke Hoekstra, Vieri Del Panta, Kilian Rapp, Natasja M. van Schoor, Stefania Bandinelli, Martijn W. Heymans, Dominique Mauger, Luca Cattelani, Michael D. Denkinger, Dietrich Rothenbacher, Jorunn L. Helbostad, Beatrix Vereijken, Andrea B. Maier, Mirjam Pijnappels

**Affiliations:** 1 Department of Human Movement Sciences, Faculty of Behavioural and Movement Sciences, Amsterdam Movement Sciences, Vrije Universiteit Amsterdam, Van der Boechorststraat 7, 1081 BT Amsterdam, The Netherlands; 2 Laboratory of Clinical Epidemiology, InCHIANTI Study Group, LHTC Local Health Tuscany Center, Firenze, Italy; 3 Department of Clinical Gerontology, Robert Bosch Hospital, Stuttgart, Germany; 4 Institute of Epidemiology and Medical Biometry, Ulm University, Ulm, Germany; 5 School of Health Sciences, Faculty of Biology, Medicine and Health, University of Manchester, Manchester, UK; 6 Manchester Academic Health Science Centre and Manchester University NHS Foundation Trust, Manchester, UK; 7 Department of Health Sciences, Vrije Universiteit Amsterdam, Amsterdam, The Netherlands; 8 Amsterdam Public Health Research Institute, Department of Epidemiology and Biostatistics, VU University Medical Center, Amsterdam, The Netherlands; 9 Department of Computer Science and Engineering, University of Bologna, Bologna, Italy; 10 Geriatric Research Unit Ulm University and Geriatric Center, Agaplesion Bethesda Hospital Ulm, Ulm, Germany; 11 Department of Neuromedicine and Movement Science, Norwegian University of Science and Technology, Trondheim, Norway; 12 Faculty of Medicine Dentistry and Health Sciences, Medicine and Aged Care, University of Melbourne, Royal Melbourne Hospital, Melbourne, Australia

**Keywords:** Functioning, Individual patient data, Middle aged, Personalised care, Preventive medicine, Active aging

## Abstract

**Background:**

Identifying those people at increased risk of early functional decline in activities of daily living (ADL) is essential for initiating preventive interventions. The aim of this study is to develop and validate a clinical prediction model for onset of functional decline in ADL in three years of follow-up in older people of 65–75 years old.

**Methods:**

Four population-based cohort studies were pooled for the analysis: ActiFE-ULM (Germany), ELSA (United Kingdom), InCHIANTI (Italy), LASA (Netherlands). Included participants were 65–75 years old at baseline and reported no limitations in functional ability in ADL at baseline. Functional decline was assessed with two items on basic ADL and three items on instrumental ADL. Participants who reported at least some limitations at three-year follow-up on any of the five items were classified as experiencing functional decline. Multiple logistic regression analysis was used to develop a prediction model, with subsequent bootstrapping for optimism-correction. We applied internal-external cross-validation by alternating the data from the four cohort studies to assess the discrimination and calibration across the cohorts.

**Results:**

Two thousand five hundred sixty community-dwelling people were included in the analyses (mean age 69.7 ± 3.0 years old, 47.4% female) of whom 572 (22.3%) reported functional decline at three-year follow-up. The final prediction model included 10 out of 22 predictors: age, handgrip strength, gait speed, five-repeated chair stands time (non-linear association), body mass index, cardiovascular disease, diabetes, chronic obstructive pulmonary disease, arthritis, and depressive symptoms. The optimism-corrected model showed good discrimination with a C statistic of 0.72. The calibration intercept was 0.06 and the calibration slope was 1.05. Internal-external cross-validation showed consistent performance of the model across the four cohorts.

**Conclusions:**

Based on pooled cohort data analyses we were able to show that the onset of functional decline in ADL in three years in older people aged 65–75 years can be predicted by specific physical performance measures, age, body mass index, presence of depressive symptoms, and chronic conditions. The prediction model showed good discrimination and calibration, which remained stable across the four cohorts, supporting external validity of our findings.

**Electronic supplementary material:**

The online version of this article (10.1186/s12877-019-1192-1) contains supplementary material, which is available to authorized users.

## Background

Ageing is typically accompanied by physical and cognitive decline, leading to limitations in activities of daily living (ADL), which jeopardise older people’s functioning, independence and quality of life [1]. To prevent early decline and preserve functioning in older people, enhancing an active lifestyle is recommended and interventions aimed at enhancing this are currently widely implemented [2]. Focusing on the young old enables the initiation of such interventions well before the onset of the decline in functioning. Therefore, identifying people at increased risk of early functional decline is essential for timely initiating of targeted preventive interventions to achieve the highest possible health gains [3–6].

Previously developed prediction models for the risk of decline in functioning in community-dwelling older people consistently revealed age, sex [7–9], and arthritis-related complaints [7, 8] as independent predictors. Other predictors observed were low physical activity levels [8], impaired cognition, hypertension, higher body mass index (BMI), poor self-rated health [7], chronic diseases, reduced muscle strength and socioeconomic status [9]. However, these previous prediction models were developed in older populations with a wide age range. Major life events, such as retirement, have shown strong effects on physical activity behaviour [10, 11], hence people around the age of retirement could be an important group for increasing an active lifestyle [11, 12]. A specific focus on people around the retirement age should reveal predictors particularly relevant for this target group for instigating timely preventive interventions. Furthermore, the follow-up period in previous studies ranged from six [7] to ten years [9], but a short-term risk prediction of limitations in (instrumental) ADL functioning is likely to prove a more relevant timeframe for individuals to commit to lifestyle changes if needed [13].

In the present study we aimed to develop and validate a clinical prediction model for the onset of functional decline at three years of follow-up in older people of 65–75 years old based on four population-based cohorts across Europe. We used a broad range of predictors, including easy-to-measure physical performance variables, to identify the most sensitive parameters.

## Methods

We conducted a study in developing and validating a clinical prediction model for the onset of functional decline at three years follow-up and reported in line with the TRIPOD (Transparent Reporting of multivariable prediction model for Individual Prognosis Or Diagnosis) statement [14].

### Study population

This study included baseline data and data from the first follow-up measurement from four on-going population-based cohort studies across Europe: Germany, United Kingdom, Italy and the Netherlands. These cohorts were selected based on the availability of the data within the PreventIT consortium [15] and availability of relevant outcome and predictor variables. Data from the four cohort studies were harmonised to allow a pooled analysis.

The Activity and Function in the Elderly in Ulm study (ActiFE-ULM) is conducted in a representative sample of 1506 German community-dwelling older people (65–90 years old) living in the greater Ulm area [16]. Included measurement cycles were conducted in 2009–2010 and 2013–2014.

The English Longitudinal Study of Aging (ELSA) is conducted in the United Kingdom and comprises a representative sample of 11,391 British older people (> 50 years old) [17]. Included measurement cycles were conducted in 2004–2005 and in 2008–2009.

The Invecchiare in Chianti study (InCHIANTI) is a cohort study from Italy. It comprises a representative sample of 1453 Italian people from two municipalities in Tuscany based on age strata [18]. Included measurement cycles were conducted in 1998–2000 and 2001–2003.

The Longitudinal Aging Study Amsterdam (LASA) is conducted in a representative sample of 3107 Dutch older people [19]. Participants were sampled from population registries in 11 municipalities in the Netherlands, based on age, sex, and level of urbanisation strata. Included measurement cycles were conducted in 1995–1996 and 1998–1999.

From all four cohort studies we included participants aged 65–75 years at baseline who reported no limitations in functional ability at baseline.

### Functional decline

The outcome is the onset of functional decline at three-year follow-up (four years in ELSA), defined as any increase (worsening) in score on self-reported (instrumental) ADL. Following prior harmonisation guidelines [20], we selected only those items that overlapped across the four cohorts to create a comparable assessment of functional decline. This resulted in a selection of two items on basic ADL [21] and three items on instrumental ADL [22]: 1) dressing and undressing; 2) sitting down and standing up; 3) using own or public transportation; 4) walking up and down a flight of stairs without resting; 5) walking outside for 400 m/for five minutes without stopping (see Additional file 1: Table S1 for details). These items have shown to be well associated with fractures [23] and recurrent falls [24]. All items were recoded into a uniform dichotomous score (0 = no limitations reported; 1 = at least some limitations reported). Participants who reported no limitations in functioning at three-year follow-up were classified as experiencing no functional decline. Participants who reported at least some limitations at three-year follow-up on any of the five items were classified as experiencing functional decline.

### Candidate predictors and missing data

Candidate predictors were measured at baseline and consisted of sociodemographic, lifestyle, clinical, and physical performance variables. We recoded variables to create uniform candidate predictors across the four datasets (see Additional file 1: Table S1 for details). Sociodemographic variables included sex, age, marital status, living status, and level of education. Lifestyle variables that were considered as candidate predictors were smoking behaviour, alcohol intake and self-reported physical activity levels. Clinical variables included BMI, mean arterial pressure (mmHg), self-reported chronic diseases, depressive symptoms (defined by the validated cutoff scores for the Center for Epidemiologic Studies-Depression scale, CES-D [25] or Hospital Anxiety and Depression Scale Depression subscale, HADS-D [26]) and cognitive status (assessed with Mini-Mental State Examination, MMSE [27] or Cognitive Function Index [28]). Physical performance variables comprised the tandem stance (seconds), five repeated chair stands (seconds), gait speed (m/s), handgrip strength (kg) and self-reported fall history in the previous year. As different test protocols were used across cohorts, values for gait speed were converted to Z-scores within each cohort before pooling the data to create comparable values.

Missing values on candidate predictors were handled by multiple imputation using the multivariate imputation by chained equations (MICE) procedure within each cohort [29], using information from all candidate predictors within the specific cohort. Based on the percentage of participants with missing data on at least one predictor (resp. 27% in ActiFE-ULM, 21% in ELSA, 23% in InCHIANTI, 14% in LASA) we created 27 datasets with missing variables imputed [30]. Rubin’s rules were applied for pooling estimates across the imputed datasets [31].

### Statistical analysis

**Fig. 1:**
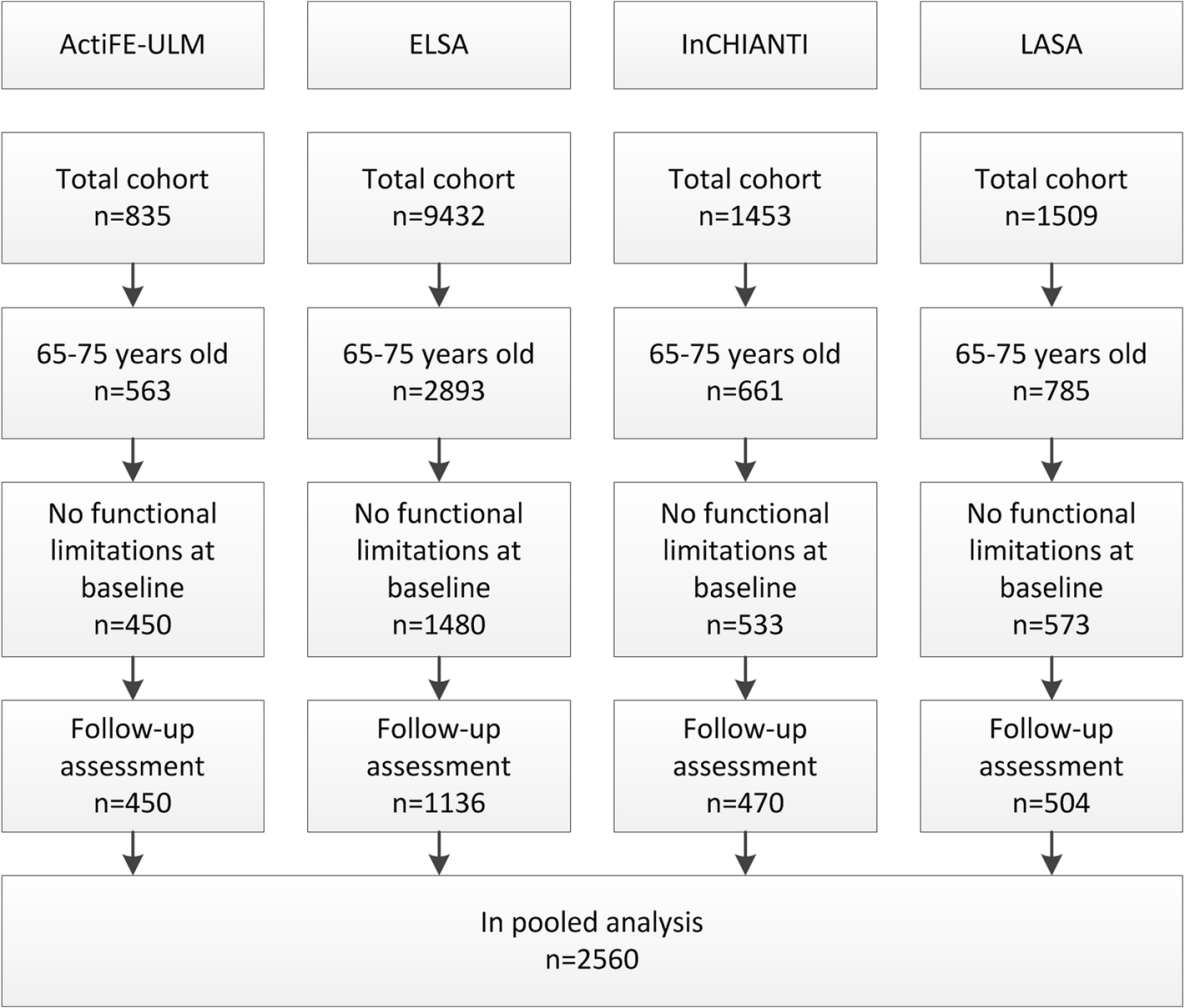
Flowchart of inclusion of participants across the four cohort studies

We combined the data from the ActiFE-ULM, ELSA, InCHIANTI and LASA cohorts in a pooled analysis to develop the prediction model (Fig. 1). For the analyses we used the rms and mice packages in R for Windows version 3.3.1 (R Development Core Team, Vienna, Austria: R Foundation for Statistical Computing).

#### Model development

The onset of functional decline was treated as a binary outcome, and logistic regression models were considered for the analysis. For all candidate predictors we fitted logistic regression models including the candidate predictor and a dummy variable as cohort index to account for different baseline risks within each cohort [32]. First, continuous predictors were examined on linearity using restricted cubic splines [33]. If the spline function indicated a non-linear association, we modelled the variable with a spline function with three knots at 10th, 50th and 90th percentile [34]. Second, we assessed multicollinearity among candidate predictors with Spearman’s correlation coefficient and considered this present if r ≥ 0.40 [35]. In case of multicollinearity, the variable with the highest predictive value was included in the multivariable model. We excluded the variable living status (multicollinear with marital status).

For developing the prediction model, we fitted a multivariable logistic regression model in the pooled dataset of four cohorts, including all candidate predictors and the dummy cohort variable. We applied a stepwise backward elimination procedure to exclude variables from the model that were not statistically significant (likelihood ratio test *p* > 0.05). Only variables with *p* < 0.05 after applying Rubin’s rules were considered significant predictors [36] and odds ratios (OR) and 95% confidence intervals (95%CI) were estimated. Performance of the model developed was assessed using the area under the receiver operating curve (C statistic, 0.50 represents no discrimination and 1.00 represents perfect discrimination) and the calibration intercept and slope (intercept of 0 and slope of 1 represent perfect calibration) [33]. Performance statistics are reported with median (interquartile range, IQR) across imputed datasets [37]. To assess the robustness of findings of the stepwise backward elimination, we performed sensitivity analyses by repeating the procedure in complete-cases (80.6% of total).

#### Internal-external cross-validation

To assess heterogeneity of findings across the cohorts and evaluate the external validity of the model, we performed an internal-external cross-validation [32, 38]. This is a novel strategy recommended for developing and validating prediction models in pooled data. Since all datasets are used for model development, all available information on the predictors is used and power is optimised [32, 38]. Through an iterative approach, this procedure assesses the external validity of the model across the four different datasets. In our study, the internal-external cross-validation consisted of the following steps: 1) Using three pooled datasets for developing the prediction model with the set of selected predictors from the stepwise backward elimination; 2) Using the remaining fourth dataset to validate the model; 3) Assessing model performance of the derivation dataset through the C statistic, calibration intercept and slope; 4) Rotating steps 1–3 across the four datasets. We compared model performance across the four iterations of the internal-external cross-validation [39].

#### Internal validation, model performance and risk scores

We performed internal validation by applying bootstrapping techniques to address the possibility of overfitting [33]. Using 250 bootstrap samples we obtained shrinkage factors and we multiplied these with the original coefficients from the developed model. We fitted a new intercept to maintain overall calibration, which resulted in our final prediction model. Model performance of the final model was assessed with the C statistic and calibration intercept and slope. We developed a clinical prediction rule for the final model to calculate an absolute risk score, based on the procedures described by Sullivan and colleagues [40]. We estimated the sensitivity, specificity, positive predictive value (PPV) and negative predictive value (NPV) of the clinical prediction rule.

## Results

### Study participants

A total of 2560 participants were eligible for inclusion in the pooled analysis (mean age 69.7 years; 47.4% females, Fig. 1 for overview). Most included participants were from the ELSA cohort (*n* = 1136, 44.4% of total). Prevalence of functional decline at three-year follow-up was comparable across cohorts, with overall 572 (22.3%) participants showing functional decline at follow-up (22.2% in ActiFE-ULM, 23.9% in ELSA, 19.4% in InCHIANTI and 21.6% in LASA). Table 1 presents descriptive characteristics of the potential predictors in the four cohorts and the pooled database. Participants in the InCHIANTI study had lower education (13.4% with > 9 years education compared to 55.8% overall) and had the fastest gait speed (mean ± SD, 1.29 ± 0.20 m/s compared to 1.01 ± 0.40 m/s overall).

**Table 1:** Baseline characteristics of 65–75 years old people from the four European cohorts

Variable	ActiFE-ULM *n* = 450	ELSA *n* = 1136	InCHIANTI *n* = 470	LASA *n* = 504	Total *n* = 2560
Outcome					
Functional decline at follow-up	100 (22.2)	272 (23.9)	91 (19.4)	109 (21.6)	572 (22.3)
Sociodemographic variables					
Sex, female	181 (40.2)	553 (48.7)	228 (48.5)	252 (50.0)	1214 (47.4)
Age, years	70.4 ± 2.8	69.4 ± 3.1	69.6 ± 3.0	70.0 ± 3.1	69.7 ± 3.0
Living alone	74 (16.4)	294 (25.9)	61 (13.0)	142 (28.2)	571 (22.3)
Married	346 (76.9)	769 (67.7)	349 (74.3)	341 (67.7)	1805 (70.5)
> 9 years formal education	237 (52.7)	895 (78.8)	63 (13.4)	234 (46.4)	1429 (55.8)
Lifestyle and clinical variables					
Smoking status					
Never smoker	230 (51.1)	478 (42.1)	238 (50.6)	138 (27.4)	1084 (42.3)
Former smoker	186 (41.3)	543 (47.8)	139 (29.6)	239 (47.4)	1107 (43.2)
Current smoker	34 (7.6)	114 (10.0)	93 (19.8)	91 (18.1)	332 (13.0)
Alcohol consumption					
Never/< 1 month	76 (16.9)	276 (24.3)	117 (24.9)	78 (15.5)	547 (21.4)
Low	119 (26.4)	412 (36.3)	169 (36.0)	247 (49.0)	947 (37.0)
Moderate	125 (27.8)	213 (18.8)	95 (20.2)	41 (8.1)	474 (18.5)
High	130 (28.9)	211 (18.6)	87 (18.5)	102 (20.2)	530 (20.7)
Physical activity					
High	141 (31.3)	265 (23.3)	39 (8.3)	165 (32.7)	610 (23.8)
Moderate	143 (31.8)	676 (59.5)	203 (43.2)	164 (32.5)	1186 (46.3)
Low	142 (31.6)	195 (17.2)	226 (48.1)	169 (33.5)	732 (28.6)
BMI, kg/m^2^	26.9 ± 3.6	27.0 ± 3.8	27.4 ± 3.8	26.3 ± 3.5	27.0 ± 3.7
Mean arterial pressure, mmHG	100.2 ± 9.7	96.6 ± 11.7	104.5 ± 11.4	104.4 ± 14.1	100.3 ± 12.3
Self-reported disease					
Cardiovascular	68 (15.1)	229 (20.2)	29 (6.2)	82 (16.3)	408 (15.9)
Diabetes	50 (11.1)	71 (6.3)	50 (10.6)	24 (4.8)	195 (7.5)
COPD	7 (1.6)	59 (5.2)	36 (7.7)	43 (8.5)	145 (5.7)
Stroke	8 (1.8)	33 (2.9)	16 (3.4)	13 (2.6)	70 (2.7)
Arthritis	198 (44.0)	288 (25.4)	66 (14.0)	172 (34.1)	724 (28.3)
Cancer	67 (14.9)	87 (7.7)	24 (5.1)	53 (10.5)	231 (9.0)
Depressive symptoms^a^	18 (4.0)	133 (11.7)	94 (20.0)	41 (8.1)	286 (11.2)
Cognitive function^b^	29 (28–30)	30 (26–33)	27 (25–28)	28 (27–29)	NA
Physical performance variables					
Unable to perform tandem stand for 10s	26 (5.8)	101 (8.9)	42 (8.9)	83 (16.5)	252 (9.8)
Chair stands, s	10.2 ± 3.2	11.3 ± 3.3	10.2 ± 2.4	11.7 ± 3.0	11.0 ± 3.2
Gait speed, m/s	1.12 ± 0.27	0.97 ± 0.26	1.29 ± 0.20	0.95 ± 0.24	1.01 ± 0.40
Handgrip strength, kg	36.1 ± 11.2	32.9 ± 10.0	33.9 ± 11.9	33.3 ± 10.2	33.8 ± 10.7
Fall in prior 12 months^c^	128 (28.4)	248 (21.8)	79 (16.8)	138 (27.4)	593 (23.2)

*BMI* body mass index; *COPD* chronic obstructive pulmonary disease

Data are presented as mean ± SD or n (%) or median (IQR)

^a^Defined by validated cutoff score for Center for Epidemiologic Studies-Depression scale [25] (in ELSA, InCHIANTI, LASA) and Hospital Anxiety and Depression Scale-Depression subscale [26] (in ActiFE-ULM)

^b^Assessed with Mini-Mental State Examination [27] (range 1–30, in ActiFE-ULM, InCHANTI, LASA) or Cognitive Function Index [28] (range 0–44, in ELSA). Tertiles in harmonised analysis

^c^Fall in prior 24 months in ELSA

### Model development

Stepwise backward logistic regression showed that 10 of 22 potential predictors were significantly associated with functional decline at follow-up (Table 2). Time to complete five repeated chair stands showed a non-linear association with functional decline and was modelled using a spline function with three knots (at 10th, 50th and 90th percentile). Table 2 reports ORs and 95%CIs of the linear and converted variable for chair stands, as these were modelled simultaneously to account for the non-linear association. Sensitivity analysis in complete-cases resulted in similar results for the stepwise backward elimination (in Additional file 1: Table S2).

**Table 2:** Final model developed in pooled data of 65–75 year old people from the four cohorts (*n* = 2560)

Predictor	Beta^a^	Odds ratio^a^	95%CI^a^	Likelihood ratio test *p*-value
Intercept ActiFE-ULM	−9.273			
Intercept ELSA	−9.285			
Intercept InCHIANTI	−9.528			
Intercept LASA	−9.440			
Sociodemographic variables				
Age, years	0.065	1.07	(1.03–1.10)	< 0.001
Lifestyle and clinical variables				
BMI, kg/m^2^	0.086	1.09	(1.06–1.12)	< 0.001
Cardiovascular disease	0.470	1.60	(1.24–2.01)	< 0.001
Diabetes	0.396	1.49	(1.06–2.09)	0.018
COPD	0.704	2.02	(1.37–2.98)	< 0.001
Arthritis	0.351	1.42	(1.14–1.77)	0.001
Depressive symptoms^b^	0.642	1.90	(1.43–2.53)	< 0.001
Physical performance variables				
Handgrip strength, kg	−0.015	0.99	(0.98–1.00)	0.002
Z-score gait speed^c^	−0.286	0.75	(0.67–0.84)	< 0.001
Chair stands, s (linear)	0.125	1.13	(1.03–1.25)	< 0.001
Chair stands, s (spline) ^d^	−0.063	0.94	(0.85–1.04)	< 0.001

*BMI* body mass index; *CI* confidence interval; *COPD* chronic obstructive pulmonary disease

^a^Optimism-corrected coefficients, with shrinkage factor 0.946–0.951

^b^Defined by validated cutoff score for Center for Epidemiologic Studies-Depression scale [25] (in ELSA, InCHIANTI, LASA) and Hospital Anxiety and Depression Scale-Depression subscale [26] (in ActiFE-ULM)

^c^Since different tests were applied in the cohorts to assess gait speed, Z-scores were calculated per cohort:

Z_ActiFE-ULM_ = (m/s–1.12)/0.27; Z_ELSA_ = (m/s–0.97)/0.26; Z_InCHIANTI_ = (m/s–1.29)/0.20; Z_LASA_ = (m/s–0.95)/0.24

^d^Beta for spline function can be applied by converting chair stands times using 10th, 50th, 90th percentiles of chair stands scores as knot locations: ((chairstand-7.73)^3^–1.73*(chairstand-10.60)^3^ + 0.73*(chairstand-14.53)^3^)/46.24. Values for the cubic terms were converted to zero if < 0

### Internal-external cross-validation

Ten significant predictors resulting from the model development were used in the internal-external cross-validation. Rotating the internal-external cross-validation across the four cohorts, performance of the developed models remained stable with a C statistic ranging from 0.691 to 0.740 (Table 3). Calibration in the large was overall good with calibration intercepts close to zero and ranging from − 0.271 to 0.135 (Table 3). The calibration slopes remained close to one across all four cohorts and indicated a slight overfitting when LASA was the validation sample with a slope of 1.215 (Table 3).

**Table 3:** Model performance in the pooled dataset and after internal-external cross-validation

	Development of model		Internal-external cross-validation							
	Apparent performance (in 4 pooled cohorts)	Optimism-corrected performance^a^ (in 4 pooled cohorts)	Development in ELSA, InCHIANTI, LASA	External validation in ActiFE-ULM	Development in ActiFE-ULM, InCHIANTI, LASA	External validation in ELSA	Development in ActiFE-ULM, ELSA, LASA	External validation in InCHIANTI	Development in ActiFE-ULM, ELSA, InCHIANTI	External validation in LASA
	*n* = 2560	*n* = 2560	*n* = 2110	*n* = 450	*n* = 1424	*n* = 1136	*n* = 2090	*n* = 470	*n* = 2056	*n* = 504
Discrimination										
C statistic^b^	0.719 (0.717–0.721)	0.719 (0.716–0.720)	0.721 (0.719–0.723)	0.698 (0.695–0.700)	0.738 (0.736–0.740)	0.691 (0.687–0.694)	0.713 (0.712–0.716)	0.720 (0.718–0.723)	0.711 (0.709–0.713)	0.740 (0.738–0.742)
Calibration										
Intercept^c^	0.000 (0.000–0.000)	0.059 (0.047–0.073)	0.000 (0.000–0.000)	0.135 (0.113--0.159)	0.000 (0.000–0.000)	− 0.271 (− 0.293- -0.263)	0.000 (0.000–0.000)	− 0.116 (− 0.144- -0.103)	0.000 (0.000–0.000)	− 0.096 (− 0.112- -0.976)
Slope^c^	1.000 (1.000–1.000)	1.053 (1.042–1.065)	1.000 (1.000–1.000)	0.949 (0.931–0.958)	1.000 (1.000–1.000)	0.757 (0.739–0.769)	1.000 (1.000–1.000)	0.966 (0.949–0.981)	1.000 (1.000–1.000)	1.215 (1.191–1.228)

Values are median (IQR)

^a^Optimism 0.049–0.054, determined by internal validation in bootstrap samples (250 samples with replacement)

^b^C statistic of 0.50 represents no discrimination and 1.00 represents perfect discrimination

^c^Intercept of 0 and slope of 1 represent perfect calibration

### Model performance

Bootstrapping showed that a uniform shrinkage factor ranging from 0.946–0.951 across the imputed datasets was needed to adjust predictor coefficients for optimism. Table 3 shows the apparent performance of the unadjusted prediction model in the four cohorts and the performance after shrinking the coefficients. After adjusting for optimism, the final model was able to discriminate between people with and without functional decline with a C statistic of 0.719 (IQR, 0.716–0.720 across imputed datasets). Calibration of the final model was excellent with an intercept of 0.059 (IQR, 0.047–0.073) and calibration slope of 1.053 (IQR, 1.042–1.065, Table 3).

### Risk scores

**Fig. 2:**
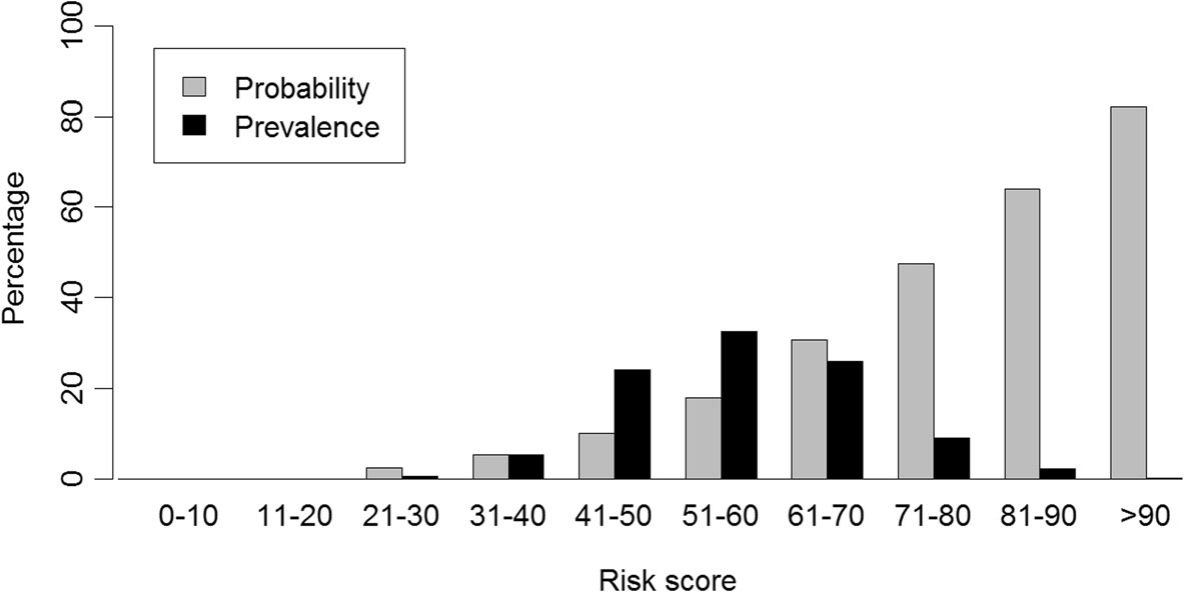
Predicted probability of functional decline by total risk scores and prevalence of the scores. Legend: Grey columns indicate the probability of experiencing functional decline at three-year follow-up with a specific risk score. Black columns indicate the prevalence of the scores within the database

Regression coefficients were converted to simple absolute risk scores to facilitate individual prediction of risk of functional decline by summing risk scores for specific characteristics (Table 4, 39]. The total score has a possible range from 0 to 117. For example, a Dutch (+ 1) female of 74 years old (+ 9), with a BMI of 23.9 (+ 0), shows no symptoms of depression (+ 0), has no cardiovascular disease or COPD (+ 0), but diagnosed with diabetes mellitus (+ 6) and arthritis (+ 5). Her handgrip strength is 18 kg (+ 7), converted gait speed is 0.214 m/s (+ 9) and she performs five repeated chair stands in 16 s (+ 17, + 0 for non-linear term). Her total risk score would be 54. Figure 2 shows the distribution of probability of functional decline across grouped risk scores and the prevalence of risk scores within the pooled database. Someone with a risk score of 54 is predicted to have a 32.7% risk of functional decline in three years and this risk applied to 17.3% of the participants in the pooled database. Predictive values for specific cutoffs in the total risk score are presented in Table 5 and illustrate an increasing specificity with increasing values for the cutoff, while this reduces the sensitivity.

**Table 4:** Score chart for calculating individual risk scores derived from the prediction model

Item	Categories	Risk score
Population (cohort)	British (ELSA)	4
	Dutch (LASA)	1
	German (ActiFE-ULM)	4
	Italian (InCHIANTI)	0
Age	65 years	0
	66 years	1
	76 years	2
	68 years	3
	69 years	4
	70 years	5
	71 years	6
	72 years	7
	73 years	8
	74 years	9
	75 years	10
Cardiovascular disease	No	0
	Yes	7
Diabetes mellitus	No	0
	Yes	6
COPD	No	0
	Yes	11
Arthritis	No	0
	Yes	5
Depressive symptoms	No	0
	Yes	10
BMI	< 25 kg/m^2^	0
	25–29.99 kg/m^2^	7
	≥30 kg/m^2^	16
Handgrip strength	≤20 kg	7
	20.01–30 kg	5
	30.01–40 kg	3
	> 40 kg	0
Z score gait speed^a^	< −1.5	17
	−1.5- −0.5	13
	-0.5-0.5	9
	0.5–1.5	5
	> 1.5	0
Time 5 repeated chair stands	≤10.7 s	0
	10.71–12.9 s	7
	> 12.9 s	17
Converted value time 5 repeated chair stands^b^	< 0.485	7
	0.485–2.091	6
	> 2.091	0
Total risk score = sum of risk scores for all items		

^a^Z-score can be calculated depending on population: Z_ActiFE-ULM_ = (m/s–1.12)/0.27; Z_ELSA_ = (m/s–0.97)/0.26; Z_InCHIANTI_ = (m/s–1.29)/0.20; Z_LASA_ = (m/s–0.95)/0.24

^b^Time of 5 repeated chair stands show a non-linear association. Converted value can be calculated with time for five repeated chair stands: ((chair stand in s-7.73)^3^–1.73*(chair stand in s-10.60)^3^ + 0.73*(chair stand in s-14.53)^3^)/46.24. Values for the cubic terms should be converted to zero if < 0

**Table 5:** Predictive value of the prediction model for different cutoffs in the total risk score

Cutoff	% in risk group	Sensitivity	Specificity	∑	PPV	NPV
≥8	99.8%	100.0%	0.2%	100.2%	22.4%	100.0%
≥16	99.1%	100.0%	1.2%	101.2%	22.6%	100.0%
≥24	92.9%	98.6%	8.7%	107.3%	23.7%	95.6%
≥32	75.8%	91.8%	28.8%	120.6%	27.1%	92.4%
≥40	51.3%	75.0%	55.5%	130.5%	32.7%	88.5%
≥48	28.6%	50.3%	77.6%	128.0%	39.3%	84.5%
≥56	11.3%	25.9%	92.9%	118.7%	51.0%	81.3%
≥64	4.0%	10.0%	97.7%	107.7%	55.3%	79.0%
≥72	0.7%	2.1%	99.7%	101.8%	66.7%	78.0%
≥80	0.2%	0.7%	100.0%	100.7%	100.0%	77.8%

*PPV* positive predicted value, *NPV* negative predictive value; *∑* sum of sensitivity and specificity

## Discussion

Based on four European cohort studies we showed that in people aged 65–75 years, the onset of functional decline in ADL at a short follow-up period of three years can be predicted by specific physical performance variables in combination with age, BMI, presence of depressive symptoms and four chronic conditions: cardiovascular disease, diabetes, COPD and arthritis. This multifactorial prediction model showed good discrimination and calibration, which both remained stable across the four cohorts in an internal-external cross validation.

Few previous studies have developed a clinical prediction model for the risk of functional decline in ADL [7–9] and those which have done so, included wide age groups within the older population (resp. 55–90+ years old [7], 40–80 years old [9] and 60–79 years old, women only [8]). The present study focused on the specific age group of 65–75 years old, since recently retired people may be a particularly relevant target group for initiating behaviour change interventions [10–12]. In the previous studies, age was consistently reported as a significant predictor [7–9] and even within our narrow age range, we found age to be a significant predictor of the onset of functional decline.

The contribution of chronic conditions in our prediction model is in line with prior models, where a higher number of chronic conditions was associated with a higher risk [9]. Of the chronic conditions, particularly arthritis seems to be an important predictor, in the broader range of older age too [7, 8]. However, the predictive effects we observed for BMI and depressive symptoms in our specific cohort were not consistently found in the studies with a wider age range [7–9]. Our findings extend the evidence on the important role of depressive symptoms in age-related decline [41] and highlight the need to consider different characteristics when screening specific age groups for risk of functional decline.

Three of the predictors identified in our model (depressive symptoms, lower handgrip strength and lower gait speed) are part of the frailty concept [42]. Frailty (which next to those three factors also encompasses unintentional weight loss and low levels of physical activity [42]) has been shown to be predictive of functional decline and mortality [43]. The prior studies on prediction models for functional decline in older people did not specifically focus on the frailty concept in the variable selection [7–9]. Den Ouden and colleagues [9] did consider physical performance variables in their prediction model. These investigators included a composite score from the short physical performance battery (SPPB) and a composite score for handgrip strength and leg extensor strength in their analysis and found only the composite muscle strength to be predictive of functional decline at ten years [9]. From our analysis it seems that the easier measure of handgrip strength alone is sufficient in predicting functional decline. Our findings emphasize the importance of considering the tests for gait speed [44–46] and chair stands [44] separately instead of a composite score like the SPPB. Moreover, our findings confirm that frailty plays an important role in the prediction of functional decline, even in this group of young older people. Future studies should consider all variables of the frailty concept as candidate predictors.

Unlike the studies by Tas and colleagues [7] and Den Ouden and colleagues [9], sex was not a significant predictor in our prediction model. This might be explained by the strong associations we observed for the physical performance measures. As physical performance measures are found to differ substantially between sexes [47], the data for handgrip strength, gait speed and chair stands may already account for the variance between males and females.

Our prediction model provides clinicians with a small set of easy-to-measure variables that discriminate well in predicting functional decline in community-dwelling people aged 65–75 years old. Clinicians can use this set of variables to screen individuals on their risk of functional decline in the coming three years. In the digital era, the presented prediction model can also be developed into an online tool that can estimate a more detailed risk score. Outcomes of the screening can help to decide whom to target for starting preventive interventions designed to reduce the risk of functional decline. Although a variety of behaviour change interventions have been developed and shown to be effective in increasing an active lifestyle in older adults [48], evidence from interventions specifically targeted towards people around the retirement age is scarce [49]. Interventions for the general population of older adults might also be suitable for the subgroup of people 65–75 years old [48], yet further investigation of interventions specifically designed for this age group is needed to optimise uptake by individuals and identify the best strategies for reducing the risk of functional decline.

This study used pooled data of four European ongoing cohort studies [16–19] to develop a prediction model specifically for a young older population. Our approach allowed the inclusion of a higher number of participants in the analysis (resulting in higher power) while at the same time assessing the generalisability of our findings across the four cohorts and enhancing external validity of the developed model in a new population [50]. Differences in baseline risk due to merging disparate samples were addressed by including cohort-specific intercepts in the model [32]. Yet, using existing data from different cohorts introduced some limitations. First, we were dependent on data available in the four cohorts and heterogeneity in measurements across the cohorts could have affected our results. Given the design of a pooled analysis, our outcome measure only included functional decline items that were available in all cohorts. Of the five included items, three addressed instrumental ADL. We expect that this might lead to a more sensitive measure in our specific cohort of adults of 65–75 years old, since it is likely that people experience decrease in instrumental ADL prior to decrease in basic ADL [9]. Although the items we used to define functional decline have shown to be a valid measure of functional performance in prior studies [23, 24], a full comparison with validated instruments to assess (instrumental) ADL is needed. Similarly, variables that were not available in all cohorts were not considered in our analysis of potential predictors. Inclusion of more sensitive variables, such as walking fast or across obstacles [51, 52], might have altered the outcomes of the stepwise backward elimination or increased the discrimination of the prediction model. Second, we restricted our analysis to people from 65 to 75 years old to focus on a target group for initiating preventive interventions [10–12, 15]. We may question whether risk identification of short-term functional decline should be expedited to an even younger age group, since 39.1% of participants of 65–75 years old in the cohort studies reported limitations on at least one ADL item at baseline (Fig. 1). The same holds for older age groups, as a large proportion of the participants included in our study were not suffering from any functional limitations after 3 years follow-up. There is a need to further investigate the onset of functional decline in adults below 65 and above 75 years of age to assess if the current model can also be applied at an earlier stage in life or if a tailored model is needed. Finally, the inclusion of one cohort that was about twice the size of the other cohorts (ELSA) might have biased the estimated predictors. To assess this potential source of bias, we applied a novel approach for developing and validating prediction models using multiple datasets, through internal-external cross-validation [32, 38]. Performing the steps of developing the model in three pooled datasets while externally validating the performance in the fourth dataset, and alternating this across the four datasets, showed consistent model performance of the predictors across the four cohorts. The small shrinkage factor further suggests that the coefficients from our prediction model are accurate in new participants. This provides strong evidence for the generalisability of our prediction model [50], although future validation in completely independent data is needed to confirm this.

## Conclusions

In people aged 65–75 years, the onset of functional decline in ADL at a short follow-up period of three years can be predicted by specific physical performance variables and age, BMI, chronic conditions and depressive symptoms. The prediction model showed good discrimination and calibration, which remained stable across the four cohort studies, supporting the external validity of our findings.

## Additional file

Additional file 1: **Table S1.** Characteristics of original variables in the four cohort studies and the harmonisation procedures. **Table S2.** Sensitivity analysis of stepwise backward procedure in complete-cases in the pooled data of 65–75 year old people from the four cohort studies (*n* = 2064). (DOCX 27 kb)

## Data Availability

According to the data agreements we signed with the steering committees of the included cohort studies of ActiFE-ULM, InCHIANTI and LASA, we are not allowed to share our data. The access to the data would need special approvals from the steering committees of all cohort studies.

## Abbreviations

ActiFE-ULM: Activity and Function in the Elderly in Ulm study
ADL: Activities of daily living
BMI: Body mass index
CES-D: Center for Epidemiologic Studies-Depression scale
CI: Confidence interval
COPD: Chronic obstructive pulmonary disease
ELSA: English Longitudinal Study of Aging
HADS-D: Hospital Anxiety and Depression Scale-Depression subscale
InCHIANTI: Invecchiare in Chianti study
IQR: Interquartile range
LASA: Longitudinal Aging Study Amsterdam
MMSE: Mini-Mental State Examination
NPV: Negative predictive value
OR: Odds ratio
PPV: Positive predictive value
TRIPOD: Transparent reporting of multivariable prediction model for individual prognosis or diagnosis
