# Supplementary material for: Development of a clinical prediction model for the onset of functional decline in people aged 65–75 years: pooled analysis of four European cohort studies
Source: BMC Geriatr. 2019 Jun 27;19:179. doi: 10.1186/s12877-019-1192-1 (PMC6595632; doi:10.1186/s12877-019-1192-1)
Supplement: Supplementary file 1 — Table S1. Characteristics of original variables in the four cohort studies and the harmonisation procedures. Table S2. Sensitivity analysis of stepwise backward procedure in complete-cases in the pooled data of 65–75 year old people from the four cohort studies (n = 2064). (DOCX 27 kb) [file 12877_2019_1192_MOESM1_ESM.docx]

**Additional file 1 – Jonkman et al.**

***Predicting the onset of functional decline in people aged 65-75 years old:***

***pooled analysis of four European cohort studies***

***Table S1:*** Characteristics of original variables in the four cohort studies and the harmonisation procedures.

***Table S2:*** Sensitivity analysis of stepwise backward procedure in complete-cases in the pooled data of 65-75 year old people from the four cohort studies (n=2064).

***Table S1:*** Characteristics of original variables in the four cohort studies and the harmonisation procedures.

| **Variable** | **ActiFE-ULM** | **ELSA** | **InCHIANTI** | **LASA** | **Harmonisation** |
| --- | --- | --- | --- | --- | --- |
| ***Outcome*** | | | | | |
| Functional decline | 10 self-reported items on the difficulty with ADL or iADL activities, scored  0 - no difficulty  1 - slight difficulty  2 – moderate difficulty  3 - large difficulty  4 - not possible  Selection of 5 overlapping items:   - dressing and undressing - sitting down, standing up from a chair - using own or public transport - walking up and down a staircase of 15 steps without resting - walking outside during 5 minutes without stopping | 29 self-reported items on the difficulty with ADL or iADL activities, scored  0 - no difficulty  1 - some difficulty  Selection of 5 overlapping items:   - dressing, including shoes and socks - getting up from a chair - using own car or van and using public transportation - climbing one flight of stairs without resting - walking 1/4 mile unaided | 14 self-reported items on the difficulty with ADL or iADL activities, scored  0 - no difficulty  1 - with difficulty, but without help  2 - only with help  3 - unable to do  Selection of 5 overlapping items:   - dressing and undressing - sitting down, standing up in bed - using own or public transportation - walking up and down a staircase of 10 steps without resting - walking outside for 400 meters | 6 self-reported items on the difficulty with ADL or iADL activities, scored:  0 - no difficulty  1 - with some difficulty  2 - with much difficulty, but without help  3 - only with help  4 - no I cannot  Selection of 5 overlapping items:   - dressing and undressing - sitting down, standing up from a chair - using own or public transport - walking up and down a staircase of 15 steps without resting - walking outside during 5 minutes without stopping | 5 overlapping items scored:  0 - no difficulty  1 - at least some difficulty  Total sum score on functional decline ranges 0-5  Dichotomised total score:  0 🡪 0  (no limitations)  1-5 🡪 1 (limitations reported) |
| ***Sociodemographic variables*** | | | | | |
| Sex | male/ female | male/ female | male/ female | male/ female | 0=male  1=female |
| Age | Years (continuous) | Years (continuous) | Years (continuous) | Years (continuous) | Years (continuous) |
| Living alone | Living alone yes/no | N people in household | Living alone yes/no | N people in household | 0=not alone 1=alone |
| Marital status | Married/ Single/ Divorced/ Widowed/Live apart | Married/  Cohabit/  Neither | Married/ Single/ Divorced/ Widowed | Married/ Single/ Divorced/ Widowed/Live apart | 0=married  1=not married |
| Education | No graduation/  9 years/  10-11 years/  >=12 years/  University | Not finished/ Never went to school/  Ended at age 14 or under/  At age 15/  At age 16/  At age 17/  At age 18/  At age 19 or older | Years of education (continuous) | Elementary not completed/ Elementary education/  Lower vocational education/ General intermediate education/  Intermediate vocational education/ General secondary education/ Higher vocational education/ College education/ University education | 0=more than 9 years  1= 9 years or less |
| ***Lifestyle and clinical variables*** | | | | | |
| Smoking | Never smoker/ Former smoker/ Smoker | Never smoker/  Ex-smoker/ Current smoker | Never smoker/ Former smoker/ Smoker | Never smoker/ Former smoker/ Smoker | 0=never  1=former  2=current |
| Alcohol use | Daily/ Multiple times per week/ Multiple times per month/ Less than 1 time per month/ Never | Almost daily/ 5-6 times per week/ 3-4 times per week/ Once-twice per week/ Once-twice per month/ Once every few months/Once-twice per year/ Not at all | Number of glasses alcohol per day, specified by type of drink (i.e., wine, beer, spirits) | Number of drinks per week | 0=none or < once per month  1=low (<=once per week OR ♂:1-10/ ♀:1-7) 2=moderate (multiple times per week OR ♂:11-20/ ♀:8-14) 3=high (daily OR ♂:>20/ ♀:>14) |
| Physical activity  (self-reported) | LAPAQ, min/week (continuous, used tertiles to create categories) | Sedentary (mild exercise 1–3 times a month, no moderate or vigorous activity)/ Low (mild, but no vigorous activity at least once a week)/ Moderate (moderate activity more than once a week, or vigorous activity between once a week to 1–3 times a month)/ High (heavy manual work or vigorous activity more than once a week) | Hardly any physical activity (low)/ Mostly sitting, some walking (low)/ Light exercise 2-4 hrs per week (moderate)/ Moderate 1-2 hrs or light >4 hrs per wk (moderate)/ Moderate exercise >3 hrs per wk (high)/ Intense exercise many times per wk (high) | LAPAQ, min/week (continuous, used tertiles to create categories) | 0=high  1=moderate  2=low |
| BMI | Kg/m^2^ (continuous) | Kg/m^2^ (continuous) | Kg/m^2^ (continuous) | Kg/m^2^ (continuous) | Kg/m^2^ (continuous) |
| Blood pressure | Diastolic & systolic blood pressure in mmHg (continuous) | Diastolic & systolic blood pressure in mmHg (continuous) | Diastolic & systolic blood pressure in mmHg (continuous) | Diastolic & systolic blood pressure in mmHg (continuous) | Mean arterial pressure (mmHg) |
| Number of medications | Number of medications | *[Not able to retrieve from data]* | Number of medications | Number of medications | NOT INCLUDED in pooled data since missing in ELSA |
| Chronic disease (self-reported) | Myocard / Heart failure | Cardiovascular conditions | Cardiovascular disease | Cardiovascular disease | 0=no  1=yes |
|  | Diabetes Mellitus | Diabetes Mellitus | Diabetes Mellitus | Diabetes Mellitus | 0=no  1=yes |
|  | COPD | Chronic lung disease | COPD | COPD | 0=no  1=yes |
|  | Stroke | Stroke | Stroke | Stroke | 0=no  1=yes |
|  | Arthritis (rheuma/osteo) | Arthritis | Arthritis (rheuma/osteo) | Arthritis (rheuma/osteo) | 0=no  1=yes |
|  | Cancer | Cancer | Cancer | Cancer | 0=no  1=yes |
|  | Neurologic | Asthma | Peripheral artery disease | Peripheral artery disease |  |
|  | Chronic kidhey | Osteoporosis |  |  |  |
|  | Chronic liver | Parkinson's disease |  |  |  |
|  | Gastric ulcer | Alzheimer's/ Dementia |  |  |  |
|  | Thyroid | Psychiatric disorder |  |  |  |
|  | Chronic pain |  |  |  |  |
| Depressive symptoms | HADS-D (continuous score 0-21, cut-off >=8) | CES-D 8 scale (continuous score 0-8, cut-off >=3) | CES-D score (continuous score 0-60, cut-off >=16) | CES-D score (continuous score 0-60, cut-off >=16) | 0=no symptoms  1=symptoms of depression (according to validated cut-off scores) |
| Cognitive functioning | MMSE score (continuous score 1-30) | Cognitive Function Index (continuous score 0-44, distribution not comparable to MMSE) | MMSE score (continuous score 1-30) | MMSE score (continuous score 1-30) | 0=high  1=moderate  2=low |
| ***Physical performance variables*** | | | | | |
| Tandem stance, eyes open | Time in s (continuous) | Did not hold semi-tandem/Held for 10s, 70 years or older/ Held for <10s, 70 years or older/ Held for 30s, below 70 years/ Held for less than 30s, below 70s/ Not attempted | No, refused/ Holds stand for < 1 sec/ Holds stand for 2-9 sec/ Holds stand for 10 sec, postural sway/ Holds stand 10 sec, no sway | Time in s (continuous) | 0=able to perform for 10s/ 1=not able to perform for 10s |
| Five repeated chair stands | Time in s for 5x (continuous) | Time in s for 5x (continuous) | Time in s for 5x (continuous) | Time in s for 5x (continuous) | Time in s for 5x (continuous) |
| Gait speed | 4m usual speed in m/s (continuous) | 8ft usual speed in m/s (continuous) | 7m usual speed in m/s (continuous) | 3+3m as fast as possible in m/s (continuous) | Z-score of m/s per cohort study (continuous) |
| Grip strength | Maximum kg 1 of 2 hands (continuous), 2 measurements | Maximum kg 1 of 2 hands (continuous), 3 measurements | Maximum kg 1 of 2 hands (continuous), 2 measurements | Maximum kg 1 of 2 hands (continuous), 2 measurements | Maximum kg of all measurements (continuous) |
| Fall history | Number of falls in last year | Number of falls in last 2 years | Number of falls in last year | Number of falls in last year | 0=no faller  1=faller |

*ADL* activities of daily living; *CES-D* Center for Epidemiologic Studies-Depression[1]; *iADL* instrumental activities of daily living; *HADS-D* Hospital Anxiety and Depression Scale[2]; *LAPAQ* LASA Physical Activity Questionnaire[3,4]; *MMSE* Mini-Mental State Examination[5].

***Table S2:*** Sensitivity analysis of stepwise backward procedure in complete-cases in the pooled data of 65-75 year old people from the four cohort studies (n=2064).

| **Predictor** | **Beta** | **Odds ratio** | **95% CI** | **Likelihood**  **ratio test**  **p-value** |
| --- | --- | --- | --- | --- |
| Intercept LASA | -9.469 |  |  |  |
| Intercept ActiFE-ULM | -9.183 |  |  |  |
| Intercept ELSA | -9.345 |  |  |  |
| Intercept InCHIANTI | -9.528 |  |  |  |
| ***Sociodemographic variables*** | | | | |
| Age, years | 0.062 | 1.06 | (1.02-1.10) | 0.001 |
| ***Lifestyle and clinical variables*** | | | | |
| BMI, kg/m^2^ | 0.094 | 1.10 | (1.07-1.13) | <0.001 |
| Cardiovascular disease | 0.467 | 1.60 | (1.19-2.12) | 0.001 |
| Diabetes | 0.476 | 1.61 | (1.08-2.36) | 0.016 |
| COPD | 0.843 | 2.32 | (1.49-3.57) | <0.001 |
| Arthritis | 0.442 | 1.56 | (1.22-1.98) | <0.001 |
| Depressive symptoms^a^ | 0.619 | 1.86 | (1.33-2.57) | <0.001 |
| ***Physical performance variables*** | | | | |
| Handgrip strength, kg | -0.019 | 0.98 | (0.97-0.99) | 0.001 |
| Z-score gait speed^b^ | -0.289 | 0.75 | (0.66-0.85) | <0.001 |
| Chair stands, s (linear) | 0.136 | 1.15 | (1.03-1.28) | 0.013 |
| Chair stands, s (spline)^c^ | -0.069 | 0.93 | (0.84-1.04) | 0.211 |

*BMI* body mass index; *COPD* chronic obstructive pulmonary disease.

^a^Defined by validated cutoff score for Center for Epidemiologic Studies-Depression scale[1] (in ELSA, InCHIANTI, LASA) and Hospital Anxiety and Depression Scale-Depression subscale[2] (in ActiFE-ULM).

^b^Since different tests were applied in the cohorts to assess gait speed, Z-scores were calculated per cohort:

Z_ActiFE-ULM_ = (m/s–1.12)/0.27; Z_ELSA_ = (m/s–0.97)/0.26; Z_InCHIANTI_ = (m/s–1.29)/0.20; Z_LASA_ = (m/s–0.95)/0.24.

^c^Beta for spline function can be applied by converting chair stands times using 10^th^, 50^th^, 90^th^ percentiles of chair stands scores as knot locations: ((chairstand-7.73)^3^ – 1.73*(chairstand-10.60)^3^ + 0.73*(chairstand-14.53)^3^)/46.24. Values for the cubic terms were converted to zero if <0.

***References Additional File 1:***

1. Radloff LS, Teri L. Use of the CES-D with older adults. Clinical Gerontology. 1986;5:119-36.

2. Bjelland I, Dahl AA, Haug TT, Neckelmann D. The validity of the Hospital Anxiety and Depression Scale. An updated literature review. J Psychosom Res. 2002;52:69-77.

3. Caspersen CJ, Bloemberg BP, Saris WH, Merritt RK, Kromhout D. The prevalence of selected physical activities and their relation with coronary heart disease risk factors in elderly men: the Zutphen Study, 1985. Am J Epidemiol. 1991;133:1078-92.

4. Voorrips LE, Ravelli AC, Dongelmans PC, Deurenberg P, Van Staveren WA. A physical activity questionnaire for the elderly. Med Sci Sports Exerc. 1991;23:974-9.

5. Folstein MF, Folstein SE, McHugh PR. "Mini-mental state". A practical method for grading the cognitive state of patients for the clinician. J Psychiatr Res. 1975;12:189-98.
